# Anti-inflammatory effects of recreational marijuana in virally suppressed youth with HIV-1 are reversed by use of tobacco products in combination with marijuana

**DOI:** 10.1186/s12977-022-00594-4

**Published:** 2022-05-31

**Authors:** Li Yin, Ashok R. Dinasarapu, Samiksha A. Borkar, Kai-Fen Chang, Kristina De Paris, Julie J. Kim-Chang, John W. Sleasman, Maureen M. Goodenow

**Affiliations:** 1 Molecular HIV Host Interaction Section, National Institute of Allergy and Infectious Diseases, 50 South Dr., Bethesda, MD 20814 USA; 2 Department of Human Genetics, Emory University, Atlanta, GA USA; 3 Department of Microbiology, University of North Carolina at Chapel Hill, Chapel Hill, NC USA; 4 Division of Allergy, Immunology and Pulmonary Medicine, Department of Pediatrics, Duke University School of Medicine, Durham, NC USA

**Keywords:** Youth with HIV-1, Marijuana, Δ9-Tetrahydrocannabinol, Cannabinoids, Tobacco, Transcriptome, Biomarkers, Bioprofiles, Inflammation

## Abstract

**Background:**

Marijuana’s putative anti-inflammatory properties may benefit HIV-associated comorbidities. How recreational marijuana use affects gene expression in peripheral blood cells (PBC) among youth with HIV-1 (YWH) is unknown.

**Approach:**

YWH with defined substance use (n = 54) receiving similar antiretroviral therapy (ART) were assigned to one of four analysis groups: YWH with detectable plasma HIV-1 (> 50 RNA copies/ml) who did not use substances (H+V+S−), and YWH with undetectable plasma HIV-1 who did not use substances (H+V−S−), or used marijuana alone (H+V−S+[M]), or marijuana in combination with tobacco (H+V−S+[M/T]). Non-substance using youth without HIV infection (H−S−, n = 25) provided a reference group. PBC mRNA was profiled by Affymetrix GeneChip Human Genome U133 Plus 2.0 Array. Differentially expressed genes (DEG) within outcome groups were identified by Significance Analysis of Microarrays and used for Hierarchical Clustering, Principal Component Analysis, and Ingenuity Pathways Analysis.

**Results:**

HIV-1 replication resulted in > 3000 DEG involving 27 perturbed pathways. Viral suppression reduced DEG to 313, normalized all 27 pathways, and down-regulated two additional pathways, while marijuana use among virally suppressed YWH resulted in 434 DEG and no perturbed pathways. Relative to H+V−S−, multiple DEG normalized in H+V−S+[M]. In contrast, H+V−S+[M/T] had 1140 DEG and 10 dysregulated pathways, including multiple proinflammatory genes and six pathways shared by H+V+S−.

**Conclusions:**

YWH receiving ART display unique transcriptome bioprofiles based on viral replication and substance use. In the context of HIV suppression, marijuana use, alone or combined with tobacco, has opposing effects on inflammatory gene expression.

**Supplementary Information:**

The online version contains supplementary material available at 10.1186/s12977-022-00594-4.

## Background

Marijuana contains a complex variety of cannabinoids, including Δ^9^-tetrahydrocannabinol (THC) and cannabidiol (CBD), known to impact inflammatory and lymphocyte activation pathways in lymphoid tissues and brain [1–4]. While marijuana continues to be classified under United States federal law as an illegal drug, medical use of cannabis is legal in at least 35 states, including 16 states and the District of Columbia where recreational marijuana is approved for individuals over the age of 21 [5]. In many states, HIV infection is the sole indication for the use of medicinal marijuana as adjuvant therapy [6]. Cannabis is effective in the treatment of HIV-associated peripheral neuropathic pain, improves appetite, and enhances overall quality of life in people with HIV [6, 7]. There is emerging evidence that marijuana use attenuates pro-inflammatory pathways in HIV-infected adults, as well as in non-human primates infected with simian immunodeficiency virus [8–10]. However, few studies have examined the effect of recreational or medicinal cannabis use on specific inflammatory pathways in peripheral blood cells (PBC) among individuals with HIV [8].

More than 27% of new HIV infections in the United States occur among youth between the ages of 20 and 24 [11]. HIV-associated chronic lymphocyte and macrophage activation drives many of the inflammatory consequences in YWH, including neurocognitive, metabolic, and vascular comorbidities, which persist even with sustained viral suppression by antiretroviral therapy [12–16]. Over a third of YWH regularly use recreational marijuana, both domestically and internationally [17, 18], yet little is known about effects of marijuana use alone, or in combination with tobacco products, on overall health and long-term outcomes in YWH [18]. The role of marijuana in modulating inflammatory pathways is particularly relevant to this population who face a lifetime of living with HIV infection and antiviral treatments.

The hypothesis for this study is that recreational marijuana use by YWH modulates PBC transcriptional pathways associated with immune activation and inflammation. YWH have not developed the extent of comorbid conditions common in older adults with HIV-1 and are an ideal population to examine the interface between viral suppression, inflammation, and substance use. Using a *computational biology* approach, unique genetic and cellular pathways within the PBC transcriptome were defined in a population of virally suppressed YWH on ART who used marijuana, alone or in combination with tobacco, compared to a reference group of youth without HIV-1 (YWOH).

## Results

### Study population

Based on substance use profiles, toxicology criteria, and viral load, 88 individuals (34 YWOH and 54 YWH) were included in the analysis (Table 1). YWOH included 25 individuals who did not use any substance (Reference Group, H–S−) and 9 who used a combination of marijuana, tobacco and alcohol (Group I, H–S+[M/T/A]). At the time of study blood draw, 8 non-substance using YWH had detectable plasma HIV-1 (Group II, H+V+S−). Among YWH, there were 46 YWH with undetectable plasma HIV-1 including: Group III, YWH who did not use any substance (n = 19) (H+V−S−); Group IV, YWH who used marijuana only (n = 8) (H+V−S+[M]); and Group V, YWH who used marijuana in combination with tobacco (n = 19) (H+V−S+[M/T]). Self-reported regular substance use by inhalation (smoking) in groups, IV and V was similar ranging from 3 to 13 years. Plasma carboxy THC (THCA) and cotinine levels, as determine by toxicology, were also similar across the respective groups. There were inadequate numbers of YWH who used tobacco alone to include in the analysis. While YWOH were younger (median age 22 years) than YWH (median age 24 years) due to the study design, there were no significant age differences among YWH groups or YWOH using or not using substances. The study population was predominantly African American and male with no significant differences among study groups regardless of HIV infection and viral status, except that YWOH using substances (Group I) had a lower proportion of African American compared with other groups. All YWH received ART for a median of 2.7 years with no significant difference between study groups. Combination ART regimens included nucleoside reverse transcriptase inhibitors (NRTIs), primarily emtricitabine and tenofovir, in combination with protease inhibitors (ritonavir-boosted atazanavir) or a non-nucleoside reverse transcriptase inhibitor (efavirenz). The types of ART were balanced across the study groups. CD4 T cell counts at end of study were similar between YWH and YWOH [13]. Nadir CD4 T cell counts were similar among YWH groups. Across all groups, median CD4 T cell counts at end of study were within the normal range [19].

**Table 1:** Demographic and clinical characteristics of study groups (n = 88)

Characteristics	Youth without HIV-1		Youth with HIV-1 receiving ART			
			VL > 50	VL ≤ 50^c^		
	Reference (n = 25)	Group I (n = 9)	Group II (n = 8)	Group III (n = 19)	Group IV (n = 8)	Group V (n = 19)
Substance use	None	Marijuana + Tobacco + Alcohol	None	None	Marijuana	Marijuana + Tobacco
Group abbreviation	H–S−	H–S+[M/T/A]	H+V+S−	H+V−S−	H+V−S+[M]	H+V−S+[M/T]
THCA conc. (ng/ml)^a^	≤ 1	118 [50, 336]	≤ 1	≤ 1	122 [65, 242]	212 [167, 322]
Cotinine conc. (ng/ml)^a^	≤ 1	34 [13, 70]	≤ 1	≤ 1	≤ 1	40 [18, 45]
Years of regular use^a^						
Marijuana	NA	4.5 [3.0, 6.0]	NA	NA	7.5 [5.8, 10.8]	7.0 [5.0, 8.0]
Tobacco	NA	4.5 [3.0, 6.0]	NA	NA	NA	7.0 [5.5, 10.3]
Age (years)^a, #^	22 [20, 23]	22 [20, 23]	24.5 [23, 25]	24 [23, 26]	23 [22, 25]	24 [22, 25]
Male (%)^†^	68	100	75	84	75	95
African American (%)^‡^	80	33	88	79	100	63
Years on ART^a, $^	0	0	2.6 [2.2, 2.9]	2.9 [2.4, 2.9]	2.6 [1.4, 2.8]	2.6 [2.5, 2.9]
ART regimen (%)^b, §^						
PIs	NA	NA	88	63	63	79
NRTIs	NA	NA	100	95	100	89
NNRTIs	NA	NA	25	37	50	32
HIV-1 RNA (copies/mL plasma)^a^	NA	NA	6959 [127, 19655]	≤ 50	≤ 50	≤ 50
CD4 Tcell count (cells/µL)^a, ¶^						
End of study	751 [462, 864]	692 [447, 873]	574 [307, 818]	736 [581, 964]	624 [522, 862]	688 [438, 850]
Nadir	NA	NA	459 [269, 626]	451 [341, 490]	625 [416, 632]	360 [285, 501]

NA: not applicable. PIs: protease inhibitors. NRTIs: nucleoside reverse transcriptase inhibitors. NNRTIs: non-nucleoside reverse transcriptase inhibitors

^a^Median [25th and 75th quartile range]

^b^Percent of YWH (n = 54)

^c^Virally suppressed YWH, who used tobacco only or combinations of other substances, were excluded from the analyses due to small sample size

^#^Ages were similar between Reference Group and Group I (p = 0.6107) and among YWH groups (p = 0.7947). YWOH was younger than YWH (p < 0.0001)

^†^Gender proportions were similar among study groups (p = 0.1611)

^‡^Proportions of African American were similar between YWOH and YWH (p = 0.3262) and within YWH groups (p = 0.1655) except that YWOH using substances had less proportion of African American in contrast to other groups (p = 0.0222)

^$^Duration on ART was similar among YWH groups (p = 0.5069)

^§^Types of ART were balanced across YWH groups (p = 0.9637)

^¶^CD4 T cell counts at end of study were similar among all groups (p = 0.7927) and nadir CD4 T cell counts were similar among YWH groups (p = 0.4208)

### Differentially expressed genes (DEG) by HIV-infection and substance use

**Fig. 1:**
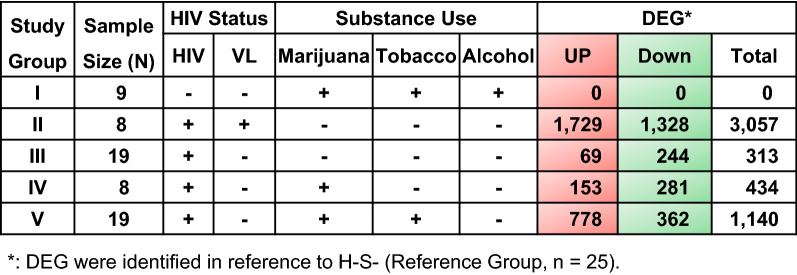
Differentially expressed genes (DEG) stratified by HIV infection and substance use. DEG analysis was performed by a pairwise comparison of the transcriptome profile between each study group (Groups I to V) to a reference group of youth without HIV-1 (YWOH) who did not use substances (n = 25). Group I (n = 9) included YWOH who used a combination of marijuana, tobacco, and alcohol; Groups II to V included youth with HIV-1 (YWH) on antiretroviral therapy (ART): II (n = 8), detectable plasma virus (+) [VL > 50 copies/ml], no substance; III to V, undetectable plasma virus (−) [VL ≤ 50 copies/ml], with no substance use [III, n = 19], marijuana use only [IV, n = 8], or marijuana combined with tobacco use [V, n = 19]. Genes showing an absolute fold change |FC| ≥ 1.3 and a permutation false discovery rate (FDR) < 0.05 were considered significantly altered in expression. Red: up-regulated DEG; Green: down-regulated DEG

Genes perturbed by HIV-infection and substance use among YWH were globally assessed, with H–S− youth (n = 25) as the universal reference group (Fig. 1). Combined use of marijuana, tobacco, and alcohol in the absence of HIV infection (H–S+[M/T/A], Group I) failed to alter gene expression compared to the reference group. In contrast, almost 5000 DEG were identified across groups of YWH. Non-substance using youth with persistent viral replication on ART (H+V+S−, Group II) had over 3000 DEG dysregulated compared to the reference group, indicating a clear effect of HIV replication on the PBC transcriptome. Non-substance using YWH virally suppressed on ART (H+V−S−, Group III) displayed about tenfold fewer DEG (n = 313), while marijuana use by virally suppressed YWH (H+V−S+[M], Group IV) resulted in 434 DEG. Down-regulation of DEG was most evident in Groups III and IV. Unexpectedly, combined use of marijuana and tobacco by suppressed YWH (H+V−S+ [M/T], Group V) resulted in 1,140 DEG, with a majority up-regulated.

### Analysis by hierarchical clustering and distribution of principal components

**Fig. 2:**
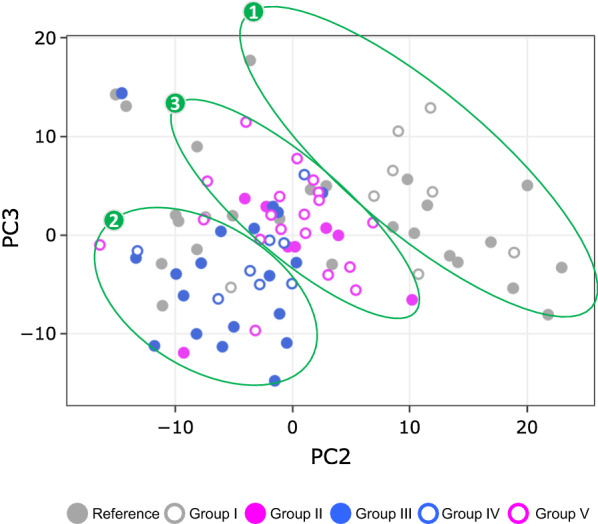
Principal component analysis (PCA). PCA based on the top 40% highly variable DEG probes (2634 of 6586) representing the top 40% highly variable unique DEG (929 of 2322) separated the 88 study participants into three clusters when using PC2 and PC3, which accounted for 12.1% or 6.1% of the total variance, respectively. Symbols: circles, participants; ellipses, participant clusters. Circle colors: gray-filled, Reference Group (non-substance using YWOH, n = 25); gray-open, Group I (YWOH using a combination of alcohol, marijuana and tobacco, n = 9); pink-filled, Group II [non-substance using YWH who had detectable plasma virus on ART (VL > 50 copies/ml), n = 8]; blue-filled, Group III [non-substance using YWH who achieved viral suppression on ART (VL ≤ 50 copies/ml), n = 19]; blue-open: Group IV (virally suppressed YWH using marijuana alone, n = 8); pink-open: Group V (virally suppressed YWH using marijuana plus tobacco, n = 19)

Two approaches were applied to visualize relatedness among all 88 individuals based on normalized gene expression values for unique DEG. Hierarchical clustering of the top 40% of highly variable unique DEG (929 of 2322) revealed no clear clustering of gene expression patterns by HIV infection, viral replication, or substance use (Additional file 1: Fig. S1). Unsupervised PCA using all gene probes did not reveal any separations by demographic factors including age, gender, and ethnicity (Data not shown). In contrast, principal components (PC), in particular PC2 and PC3, best separated study groups into three clusters (Fig. 2). Cluster 1 consisted exclusively of YWOH, including about half of H–S− (Reference Group) and two-thirds of H–S+[M/T/A] (Group I). Cluster 2 included predominantly virally suppressed YWH, including 68% of H+V−S− (Group III) and 63% of H+V−S+[M] (Group IV). Cluster 3 included 88% of H+V+S− (Group II), as well as 84% of H+V−S+[M/T] (Group V). Overall, YWOH were distinct from YWH; among YWH, viral suppression and marijuana use clustered together, while continued viral replication clustered with dual use of marijuana with tobacco.

### Pathways perturbed by HIV-infection and substance use

**Fig. 3:**
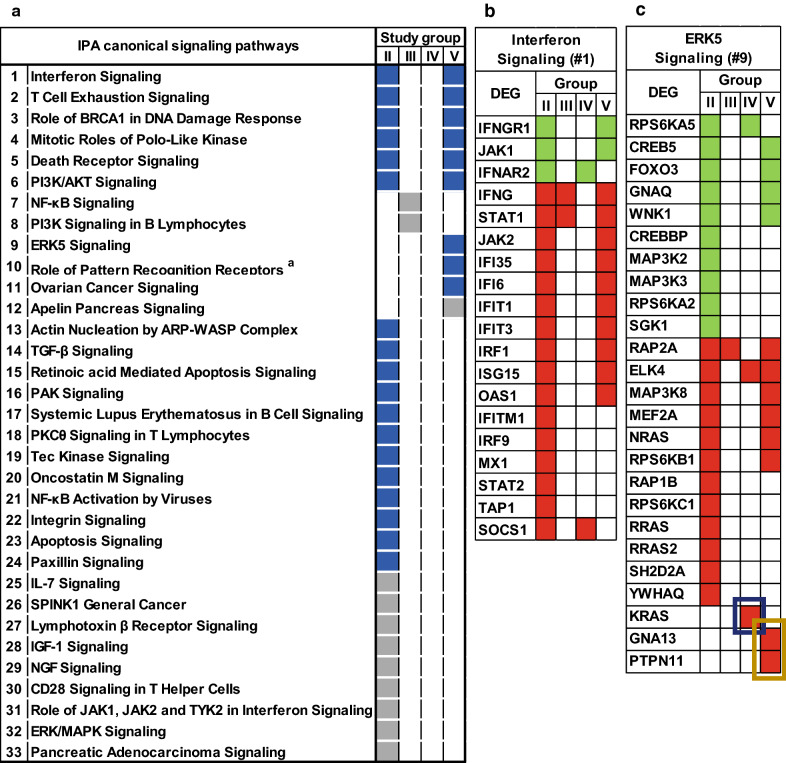
Significantly perturbed pathways in study groups and DEG within Interferon and ERK5 signaling pathways. **a** Significantly perturbed pathways were based on Ingenuity Pathway Analysis (IPA) using p ≤ 0.001 and Z score ≥ + 1 for activation (blue-filled) or p ≤ 0.001 and Z score ≤ -1 for suppression (grey-filled). VL^+^, > 50 viral copies per ml plasma; VL^−^, ≤ 50 viral copies per ml plasma. Study groups: II, VL^+^ no substance; III—V, VL^−^ with III, no substance; IV, marijuana only; and V, marijuana plus tobacco. ^a^ Pathway full name: #10, Role of Pattern Recognition Receptors in Recognition of Bacteria and Viruses. **b**, **c** DEG within the Interferon [#1] and the ERK5 [#9] Signaling Pathways (down-regulated, green-filled; up-regulated, red-filled). Boxes: blue, DEG specific for Group IV; brown, DEG specific for Group V

The sets of DEG from each group were evaluated by Ingenuity Pathway Analysis (IPA) software to identify canonical signaling pathways significantly enriched by the DEG. Among non-substance using YWH with active viral replication (Group II), a large number of DEG contributed to 27 perturbed pathways (18 activated and nine down-regulated) relative to the reference group (Fig. 3a). Control of viral replication among YWH (Groups III, IV, and V) reduced the total number of perturbed pathways to twelve (Fig. 3a). In the absence of substance use (Group III), all 27 signaling pathways perturbed by viral replication were normalized, while two additional pathways, NF-kB Signaling and PI3K Signaling in B lymphocytes, were uniquely suppressed. Marijuana use by virally suppressed YWH (Group IV) resulted in no significant perturbation of any pathways relative to the reference group. In contrast, marijuana used in combination with tobacco by virally suppressed YWH (Group V) was associated with ten dysregulated pathways, including four pathways uniquely dysregulated (three activated and one suppressed) and six pathways shared by YWH with viral replication but no substance use (Group II).

Although viral suppression with or without substance use reduced the number of perturbed pathways classified by IPA, varying numbers of genes within a pathway might remain differentially expressed. For instance, the Interferon Signaling pathway, perturbed in Groups II and V with 19 and 12 DEG, respectively, was unperturbed in Groups III and IV, even though two genes were differentially expressed (Fig. 3b). Similarly, the ERK5 Signaling pathway, perturbed in Group V with 12 DEG, was not perturbed in Group II even with 22 DEG (Fig. 3c). Within the groups, multiple DEG were identified even when the pathways were unperturbed by IPA (Fig. 3 and Additional file 1: Fig. S2A and S2B). Within each pathway, unique patterns of DEG were evident among viral and substance use groups.

#### DEG profiles affected by viral replication and substance use

**Fig. 4:**
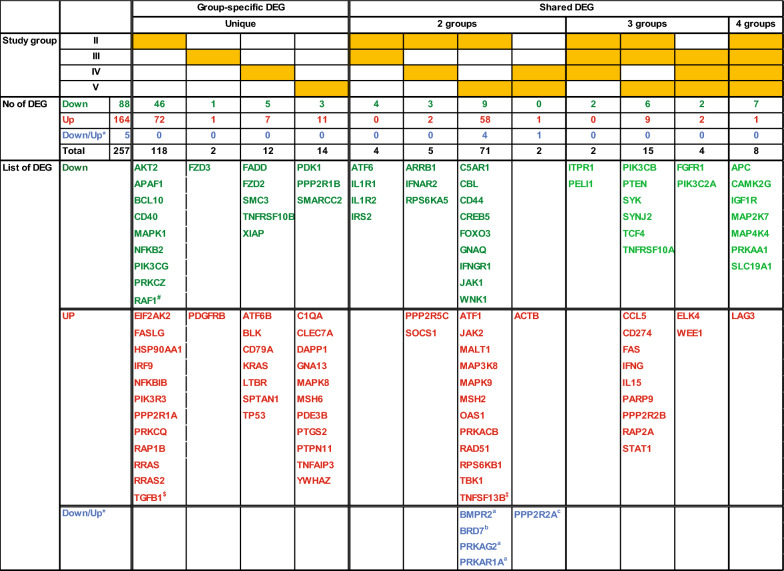
DEG in 12 pathways unique or shared by study groups. Orange-coding classifies DEG unique to or shared by study groups with down-regulated DEG texted green, up-regulated DEG texted red and DEG expressed in opposite directions in two study groups texted blue. #: Additional genes not shown CREBBP, FOXO1, IL1A, MAP3K3, NFAT5, PDPK1 and TLR6 in 2 pathways, and all others in a single pathway, including ACVR1B, ARID2, BCL6, BID, CFLAR, CSNK2A1, EP300, FOXP1, GAB2, IGF2R, IL4R, IL6R, LIMS1, LTB, MAP3K2, MDM2, MGAT5, MMP9, NAIP, PGF, POU2F1, PPP3CA, PTPRC, RAC1, RPS6KA2, SGK1, THEM4, TNFRSF21, TNFRSF25 and TNFSF14. $: Additional genes not shown NFATC2, STAT2, TGFBR3, TLR3 and YWHAQ in 2 pathways, and all others in a single pathway, including ACTA2, ANAPC4, ANAPC10, ATF5, BATF, BIRC2, BMPR1A, CAMK2D, CD28, CD81, CDKN2A, CYCS, E2F7, E2F8, EIF2S1, EIF4E, FANCA, FANCF, FANCM, FGFR2, FYN, HLA-DQA1, HLTF, IFITM1, IL10RA, IL12RB1, ILK, INPP5F, IRF3, IRF7, KIF11, LCK, MRE11, MX1, PARP11, PARP15, PARP2, PARP3, PDCD1, PLEKHA3, PLK4, PPP3CB, PRKAB2, PTTG1, RBBP8, RFC5, RPS6KC1, SH2D2A, SMAD3, SMARCE1, SOS1, STAT4, TAB3, TAP1 and TBX21. ‡: Additional genes not shown ATF3, ATR, AZI2, BARD1, BRIP1, C1QB, CASP3, CASP7, CCNB1, CCNB2, CDC7, CDC20, CDK1, CTLA4, DDX58, EOMES, FANCL, FBXO5, FZD1, HLA-DPA1, IFI35, IFI6, IFIH1, IFIT1, IFIT3, IRF1, ISG15, ITGA4, ITGB1, MEF2A, NBN, OAS2, OAS3, PARP12, PARP14, PDIA3, PIK3AP1, PRC1, PRDM1, PRKCH, RAD50, RFC3, ROCK1, SLK and UBE2N in a single pathway. *: Same gene which was up (↑)- and down (↓)-regulated in different groups. a: ↓ in II, **↑**in V. b: ↑in II, ↓in V. c: ↓ in IV, ↑ in V

To establish transcriptome profiles of YWH based on viral status and substance use, DEG within 12 perturbed pathways were aggregated across groups of YWH and de-replicated, resulting in a list of 257 unique DEG, which were then reassigned to each study group (Fig. 4). Among 257 DEG from the twelve pathways, over half (146/257) were specific to one of the groups of YWH (Groups II–V). Most DEG (118/146) were specific to Group II, YWH with active viral replication, while virally suppressed Groups III, IV, and V had only 2, 12, and 14 specific genes, respectively. The remaining 111/257 DEG were shared to varying extents among the study groups. Only eight DEG were shared among all four study groups. DEG sharing among three study groups varied but surprisingly 15 DEG shared by Groups II, III, and V were not dysregulated in YWH who used marijuana alone (Group IV). A pairwise comparison between different study groups showed few shared DEG between Groups II and III, or II and IV. Groups IV and V shared up-regulated ACTB gene involved in cell motility, structure, integrity and intercellular signaling, while PPP2R2A, a Ser/Thr phosphatase implicated in control of cell growth and division, was dysregulated in opposite directions in these study groups. Groups II and V showed a striking high number of shared DEG.

The effects of substance use on cellular and inflammatory pathways in YWH were evident when examining individual patterns DEG unique to Group III, Group IV, and Group V (Fig. 4). Non-substance using youth who achieved long-term viral suppression (Group III) showed only two unique DEG. In contrast, marijuana users (Group IV) had 12 unique DEG including down-regulated genes involved in cell death and apoptosis (FADD, TNFRSF10B and XIAP) and up-regulated genes involved in cellular proliferation (KRAS and TP53), B cell signaling (BLK and CD79A), and TNF signaling (LTBR). Similarly, YWH who used marijuana in combination with tobacco (Group V) displayed down-regulated DEG involved in negative control of cell growth (PDK1 and PPP2R1B) and up-regulated genes involved in cellular proliferation (MAPK8 and PTPN11), DNA repair (MSH6), as well as DEG in complement, prostaglandins, innate immunity, and inflammation (C1QA, CLEC7A, PTGS2, and TNFAIP3).

Examining DEG shared by groups also revealed the effect of substance use among virally suppressed YWH. The most striking observation is the vast number of pro-inflammatory genes shared exclusively by Group II (H+V+S−) and Group V (H+V−S+[M/T]). Comparing suppressed YWH who only used marijuana (Group IV) to all other groups (Group II, III, and V) revealed normalization of unique DEG associated with marijuana use alone. These genes include down-regulated genes involved in cellular proliferation and ontogenesis (PIK3CB, PTEN and TNFRSF10A) and several genes known to be up-regulated by HIV infection including CCL5, CD274 (PD-L1), FAS, IFNG, IL-15 and STAT1. These results support a specific effect of marijuana on the PBC transcriptome in the context of viral suppression. There were five genes BMPR2, BRD7, PRKAG2, PRKAR1A and PPP2R2A dysregulated in opposite directions between Group V (H+V−S+[M/T]) and Group II or Group IV suggesting an association with tobacco use.

**Fig. 5:**
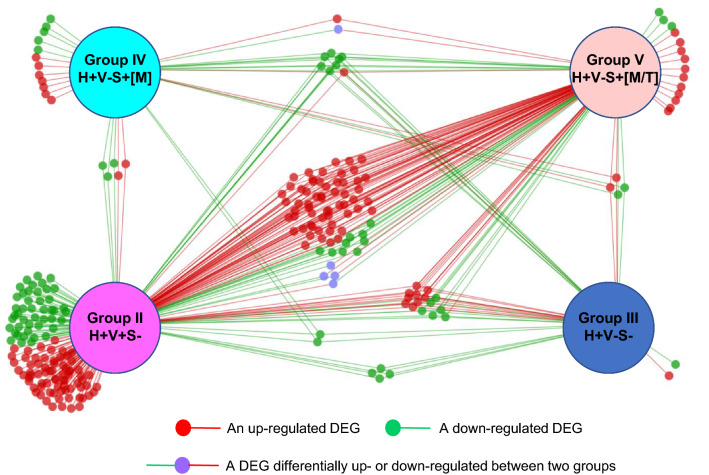
Relationship of unique DEG in 12 pathways across study groups. DEG in each of 12 pathways including 6 pathways perturbed in both Group II and Group V, two perturbed uniquely in Group III, and 4 perturbed specifically in Group V, were collected, deduplicated and assigned back to study groups. The relationships of DEG across groups were displayed as networks to display DEG unique to each group or shared between/among groups. Red dot/line, an up-regulated DEG; green dot/line, a down-regulated DEG; purple dot with green and red lines, differentially up- or down-regulated between two groups

Figure 5 is a network display of the 257 unique DEG outlined in Fig. 4 showing gene expression profiles unique to study groups, including DEG specific to each group or those shared with other one or more groups. Distinct gene expression profiles among H+V+S− (Group II), H+V−S− (Group III) and H+V−S+[M] (Group IV) indicate remarkable gene regulation by viral suppression alone or in combination with marijuana use. Massive gene sharing between H+V+S− (Group II) and H+V−S+[M/T] (Group V) point to inflammatory effect of tobacco used in combination with marijuana in virally suppressed YWH.

## Discussion

Putative anti-inflammatory properties of cannabinoids have led to increased medicinal marijuana use among the general public [8, 20–22]. Many studies of substance use effects in HIV-associated inflammatory pathways involve older, chronically infected adults using multiple substances, including cocaine, opioids, tobacco, alcohol, and methamphetamine, in combination with marijuana [23–27]. While there is evidence supporting the anti-inflammatory effects by marijuana use, polysubstance use presents challenges in identifying marijuana-specific effects on immune pathways [9, 27–29].

Our study focused on a unique population of YWH with few comorbidities who initiated ART prior to CD4 T cell decline, displayed sustained viral suppression for 3 years on ART, and showed evidence of chronic inflammation even with sustained viral suppression [12–14]. YWH who used substances self-reported regular use over the previous 3 to 13 years, with recent use validated by toxicology. The study design enables a detailed assessment of the global inflammatory transcriptome in YWH with or without substance use compared to a non-substance using reference group of YWOH, balanced for age, gender, and race [13, 14, 17, 18, 30]. Importantly, the study population, composed primarily of young African American males who use no substance or use recreational marijuana alone or in combination with tobacco [18, 31], reflects the predominant demographic of current new infections in the United States.

Unsupervised analysis of PBC transcriptome profiles revealed segregation between YWOH and YWH groups based on HIV replication and substance use profiles. Persistent low level viral replication while receiving ART significantly altered multiple DEG, while viral suppression to undetectable levels modulated this effect. More importantly, marijuana used alone or in combination with tobacco had substantial effects on the transcriptome profile even when viral replication was suppressed by ART. Compared to the reference group of non-substance using YWOH, virally suppressed YWH using marijuana alone displayed an anti-inflammatory profile, while use of marijuana plus tobacco resulted in a distinct pro-inflammatory profile. The pro-inflammatory profile displayed by YWH using marijuana combined with tobacco may stem from interactions between marijuana and tobacco, and/or a dominant modifying effect by tobacco on inflammatory pathways. Unfortunately, our cohort did not include adequate numbers of YWH who used tobacco alone to examine a tobacco-only effect on the transcriptome. Nonetheless, a key finding of our study is the profile associated with marijuana and tobacco co-use by virally suppressed YWH, which resembled, in part, the profile of non-substance using YWH with persistent viral replication. This observation may have grave implications for YWH who continue to regularly use tobacco with or without concomitant marijuana use and face a lifetime of living with HIV. Tobacco use among YWH may increase the long-term metabolic, cardiovascular, and neurocognitive complications of living with HIV [14–16, 32, 33]. These results suggest that significant efforts are needed to mitigate tobacco use among YWH who otherwise display viral control and maintain normal CD4 T cell counts. In contrast, the anti-inflammatory effects by marijuana use alone could limit viral replication and sustain viral latency, perhaps by inhibition of T cell activation or attenuation of macrophage infection [27, 34, 35]. Combined substance use including tobacco, marijuana, and alcohol by YWOH produced no discernable perturbations of gene expression. Ex vivo studies using human PBMC and transcriptome analysis from adults without HIV who heavily used tobacco (> 10 cigarettes per day) revealed upwards of 100 DEG [36, 37]. In contrast, our studies of younger individuals without HIV, who did not use tobacco heavily, displayed no effect on the PBC transcriptome by marijuana, tobacco, and alcohol, perhaps due to lower cumulative tobacco exposure or the polysubstance use of marijuana plus tobacco and alcohol.

Assessment of multiple genes and relevant canonical pathways among various YWH outcome groups provides mechanistic insights of the effects by HIV replication and substance use on the global PBC transcriptome. Most of the 27 pathways perturbed by HIV replication involve immune response and inflammatory pathways, with metabolic and cell cycle pathways affected as well. These results support previous gene set enrichment studies using either whole blood or selected cell populations in people with HIV, which all revealed perturbed gene signatures associated with HIV replication, disease progression, and clinical outcomes [38–41]. In contrast, viral suppression by ART among non-substance using YWH significantly reduced gene perturbations [42], providing an opportunity to examine how marijuana or marijuana used in combination with tobacco affects the transcriptome in the absence of viral replication. Marijuana use alone resulted in altered regulation of multiple unique genes not observed in the other groups of virally suppressed YWH. Unique DEG, particularly downregulation of FADD and upregulation of KRAS (RAS) and TP53 have been implicated in cannabinoid signaling in animal models and ex vivo cultured cells lines indicating a cannabis effect on cellular proliferation and apoptotic pathways [43–49]. Furthermore, comparing genes perturbed among non-substance using virally suppressed and unsuppressed YWH, as well as suppressed YWH using marijuana plus tobacco to the group of virally suppressed YWH using only marijuana, revealed marijuana specific effects.

Unique normalized DEG seen solely in the marijuana group included CCL5, CD274 (PDL-1), FAS, IFNγ, IL-15 and STAT1, genes in pro-inflammatory pathways known to be perturbed by HIV infection [50–56]. THC has an anti-CCL5 effect in SIV-infected rhesus macaques and *Cannabis sativa* is postulated to block signaling through Jak/Stat [57–59]. Our study is the first observation that marijuana may affect signaling through IL-15 and CD274 (PD-L1) in individuals with HIV. While IL-15 has been shown to increase HIV replication, use of IL-15 as a possible therapy to reverse latency for HIV infection has also been suggested [60–63]. THC mediates anti-inflammatory effects on IL-15 and IFNγ in macrophages in vitro [64], but marijuana effects on these pathways in the context of HIV have not been explored. PD-1 (CD274) has long been known to be upregulated on T cells of HIV-infected individuals and is positively correlated with viral load to induce cellular anergy with associated declines in cellular proliferation, cytotoxic function, and cytokine secretion [65–67]. If THC inhibits CD274 (PD-L1) expression in HIV, similar to pancreatic cancer studies [68], then inhibition of PD-1/PD-L1 by marijuana might reverse HIV-mediated T cell exhaustion [65–67].

Among the DEG perturbed in suppressed YWH who used marijuana in combination with tobacco, multiple pro-inflammatory genes associated with tobacco use were identified. For example, BMPR2, BRD7, PRKAG2, PRKAR1A and PPP2R2A (PP2A Bα subunit B55α), which are differentially expressed between group V (H+V−S+[M/T]) and groups II/IV (H+V+S−/H+V−S+[M]), play roles in tobacco-mediated lung diseases and lung cancers [69–76]. Group V-specific genes PTPN11 (Shp2) is involved in acute cigarette smoke-mediated lung inflammation by increasing IL-8 production [75], while MAPK8 (JNK1) plays a key role in cigarette smoke exposure-mediated cell death [76]. Transcriptome analysis alone is insufficient to determine the precise impact of substance use on inflammation. However, the blood transcriptome derived primarily from leukocytes offers an opportunity to examine the effects of HIV in vivo [77]. Marijuana contains multiple cannabinoids that primarily influence signaling through G protein-coupled receptors involving diverse pathways which ultimately results in the wide array of DEG, and pathways perturbed by tobacco use in YWH [78, 79].

Although the Affymetrix microarray provides a restricted survey of expressed genes in PBC, our results show clear effects by control of viral replication and substance use. In general, unsupervised analysis exposed differences between YWOH and YWH that could reflect an effect of viral infection or ART on the blood transcriptome. The regimen and percent of YWH receiving PIs, NRTIs and NNRTIs were similar across YWH groups to balance out the potential influence of ART on gene expression. One omics approach examining the effect pre-exposure prophylaxis (PrEP) in individuals without HIV found limited global effects on host gene expression with no differentially expressed genes in blood [80]. Our study was not designed to evaluate anti-inflammatory effects of marijuana in the context of a controlled clinical trial. While the routes of drug administration and total exposure to marijuana and tobacco were similar within the study cohorts, the effect of total exposure to drug, drug metabolites, or combustion products were beyond the scope of the study design. Controlled studies with clear clinical endpoints using self-administered or prescribed marijuana or isolated compounds including the use or THC or CBD are in progress [34]. However, our results do provide insight into “real life” use of recreational marijuana among YWH. The current national climate to legalize recreational and medical use of cannabinoids highlights the need to evaluate the effects of marijuana in people with HIV as these substances could have effects on inflammatory and immune pathways, as well as HIV latency. Even with optimal viral suppression, YWH will have chronic inflammation across their life span with its associated comorbidities. If it can be demonstrated that marijuana attenuates pro-inflammatory cytokines in chronic infections such as HIV, or even COVID-19 [81], our results may have implications for management of other inflammatory conditions.

## Conclusions

YWH receiving ART display unique transcriptome bioprofiles based on viral replication and substance use. Multiple DEG and pathways are perturbed by uncontrolled viral replication. In the context of HIV suppression, marijuana use alone by YWH normalizes multiple genes and pathways resulting in distinct DEG patterns. In contrast, marijuana use in combination with tobacco has opposing effects on inflammatory pathways and gene expression, as many DEG modulated by HIV replication are also perturbed by the use of marijuana plus tobacco. Substance use has an overall effect on YWH receiving ART that is likely to have long-term implications in a population who faces a lifetime of living with HIV infection and treatment.

## Materials and methods

### Demographics, clinical characteristics, and substance use among study participants

The study cohort consisted of 129 YWH infected via sexual transmission, ages 18 to 28 years, enrolled in Adolescent Medicine Trials Network (ATN) for HIV/AIDS Interventions protocol 071/101, *Assessment of Inflammatory Markers Associated with Neurocognitive Impairment in HIV-infected Adolescents.* This 3-year longitudinal study took place at 22 metropolitan sites throughout the United States and Puerto Rico (ClinicalTrials.gov Identifier NCT00683579). Completed in 2013, the primary outcome results of this 3-year study have been reported [13, 14, 18, 82]. Participants were monitored at regular intervals, at entry, and weeks 24, 48, 96 and 144 for viral load (VL), CD4 T cell counts, clinical status, antiretroviral medications, and substance use. All YWH received ART, including 64.8% using protease inhibitor-based ART, 27.8% non-nucleotide reverse transcriptase inhibitor-based ART, 1.9% Integrase Strand Transfer Inhibitor, and 5.6% received non-nucleotide reverse transcriptase inhibitor plus protease inhibitor ART. The classes and combinations of ART were similar among YWH study groups. All participants provided consent for a single blood draw obtained at the end of study (week 144) for transcriptome analysis. In addition, consent was obtained for a reference group of 55 YWOH balanced for gender and race with YWH as previously described [13]. This study was performed under protocol, *Consequences of Marijuana Use on Inflammatory pathways in HIV-Infected Youth,* reviewed and approved by the institutional review board (IRB) of Duke University and Molecular HIV and Host Interaction Section (MHHIS) at National Institute of Allergy and Infectious Diseases (NIAID), National Institutes of Health (NIH).

Use of alcohol, marijuana, tobacco products, and other substances was evaluated in two ways: Audio Computer-assisted Self-administered Interviewing (ACASI) with Alcohol, Smoking and Substance Involvement Screening Test (ASSIT) and plasma toxicology screening. ASSIST was developed for WHO [83] and was validated for adults [84] as well as adolescents [85]. ACASI reports captured the age of the participants when they first began using substances as well as the numbers of years of regular use defined as daily, weekly, or monthly use. There was a significant concordance between the self-reported substance use and toxicology results [18]. Toxicology screening was performed on plasma samples by Immunalysis Corporation (Pomona, CA; Immunalysis.com) using enzyme-linked immunosorbent assays (ELISA) for 27 analytes, including over the counter, prescription, and illicit drugs. The ELISA for marijuana detects > 1 ng/ml of plasma carboxy THC (THCA), a precursor of THC, within 1 day (acute) as well as up to 30 days of marijuana use (chronic) [86–88], while the assay for tobacco detects > 1 ng/ml of plasma cotinine, a nicotine metabolite, up to 7 days after use. Substance use was defined as detectable plasma THCA or cotinine with self-reported regular use in the past 3 months.

### Sample collection, RNA isolation, amplification, and microarray

Whole blood samples were collected in PAXgene Blood RNA Tubes (Becton, Dickinson and Company, Franklin Lakes, New Jersey, US) and stored at − 80 °C [77]. Intracellular total RNA was isolated using PAXgene Blood RNA Kit (PreAnalytiX, Hombrechtikon, Switzerland). Globin mRNAs were depleted using GLOBINclear Kit (Ambion, Waltham, MA, USA). RNA (100 ng) was amplified and labeled using GeneChip 3’ IVT Express Kit (Affymetrix, Waltham, MA, USA) and hybridized to GeneChip Human Genome U133 Plus 2.0 Array with 54,675 probes (Affymetrix, Waltham, MA, USA) in the Interdisciplinary Center for Biotechnology Research at University of Florida. Raw images in CEL format were generated with Affymetrix GeneChip Operating Software. The quality of each array was determined by manually checking mean values, variances, and paired scatter plots. All arrays passed the quality check. For downstream computational analysis statistical environment of R version 3.6 was used [89].

### Analysis plan

Study groups with a minimum of eight individuals per group to ensure reliability of analysis were defined based on HIV-1 status, VL, and substance use profile. The reference group included 25 YWOH using no substance. An additional group of 9 YWOH using a combination of alcohol, marijuana and tobacco were also included as a comparison group (Group I). There were insufficient numbers of YWOH who only used tobacco or marijuana to include in the analysis. Four study groups included ART-treated YWH: Group II, 8 non-substance users with detectable virus; Group III, 19 virally suppressed non-substance users; Group IV, 8 suppressed marijuana users; and Group V, 19 virally suppressed marijuana plus tobacco users (Table 1). YWH who used substances other than tobacco or marijuana were excluded from the analysis, as well as groups using tobacco or alcohol alone due to small sample size.

### Microarray expression normalization

Microarray raw probe signal values were corrected for background, normalized by quantile, and summarized using the Robust Multi-Array Averaging (RMA) algorithm [90]. All expression values were converted to a log_2_ scale for downstream analysis.

### Differential expression analysis

Differentially expressed genes (DEG) were identified in reference to H–S− (Reference Group, n = 25) by Significance Analysis for Microarrays (SAM), which used one-thousand permutations of the repeated measurements to estimate the percentage of genes identified by chance, the false discovery rate (FDR) [91]. Fold change (FC) was calculated for each gene by first calculating the average expression value for each study group, and then taking the anti-log_2_ of the difference between a study group and the reference group. Genes showing an absolute FC ≥ 1.3 and FDR < 0.05 were classified as DEG and used in hierarchical clustering and principal component analysis to predict the relatedness of study groups among the study cohort [39, 92]. For FDR controlled microarray analysis based on two-class unpaired test required to detect a 1.3-fold change in expression level, a samples size of 8 could provide adequate statistical power [93, 94].

### Hierarchical clustering analysis (HCA) and principal component analysis (PCA)

Both unsupervised HCA and PCA were performed using probes associated with DEG. The coefficient of variation (CV) was computed from the normalized gene expression values. The probes for each individual were ranked by CV, and the top 40% (2634 of 6586) of probes representing the top 40% of highly variable unique DEG (929 of 2322) were used for both HCA and PCA (Additional file 2). Ward.D2 method was used for HCA and heatmap was plotted using pheatmap [95]. PCA was carried out using R package, and Plotly [96] was used for graphical display and interpretation [97, 98]. Collectively, the first three principal components (PC1, PC2 and PC3) included 40.7% of the total variance with PC1 accounting for 22.5% of variance, and PC2 and PC3 accounting for 12.1% and 6.1%, respectively. To address influences by confounding demographic differences including age, gender, and ethnicity, unsupervised PCA were performed using the top 10% ranking normalized expression values of the total 54,675 gene probes.

### Pathway enrichment analysis

DEG in each study group were imported into Ingenuity Pathway Analysis (IPA) software (Qiagen; Bioinformatics, Redwood City, CA, USA) to identify canonical signaling pathways associated with genes. In IPA, canonical pathways were named by the IPA content team based on the compendium of information used to describe the functions. Fisher’s Exact Test was used to calculate the p-value by comparing the proportion of study genes associated with a particular pathway to the proportion of genes expected with p ≤ 0.0001 defining a significantly enriched pathway. The z-score was calculated by IPA to infer the activity pattern (activation or inhibition) of the canonical pathway based on the direction of the expression pattern (up-regulation or down-regulation) of the study DEG in comparison to the expected directions of same genes in the Ingenuity Knowledge Base [99]. In this study, pathways with p ≤ 0.0001 and Z score ≥ 1 were classified as activated, and those with p ≤ 0.0001 and Z score ≤ -1 were defined as suppressed [100].

### Specific DEG profile within perturbed pathways effected by viral replication and substance use

DEG in each of 12 pathways, including six pathways perturbed in both Group II and Group V, two pathways specifically perturbed in Group III, and four pathways specifically perturbed in Group V, were aggregated, de-duplicated and assigned back to each study group to identify DEG specific for each study group, or shared between or among groups. The gene relationships among groups were displayed as a network constructed using Cytoscape v3.8.0, an open source software for visualizing complex networks and integrating these with attribute data [101]. GeneCard served as the source of gene function(s) [102].

### Statistical analysis

The t test was used to compare data between two groups. One-way ANOVA was used to compare plasma concentrations of THCA and cotinine, age, ART duration, CD4 count and nadir CD4 count among groups. Chi-square test was used to predict influence of gender and race on HIV-infection or viral load status in HIV-infected individuals as well as balance in percent use of ART among study groups in YWH. GraphPad Prism 7.04 (GraphPad Software, San Diego, CA, USA) was used for all statistical analyses. Statistical significance was defined as p < 0.05.

## Supplementary Information

**Additional file 1. ** Additional Figures S1 and S2.

**Additional file 2. ** Log_2_ expression values of top 40% DEG used in hierarchical clustering analysis in Figure S1.

## Data Availability

The data was uploaded to dbGaP [103] and will released once manuscript got published.

## Abbreviations

PBC: Peripheral blood cells
YWH: Youth with HIV-1
ART: Antiretroviral therapy
H+V+S−: YWH with detectable plasma HIV-1 who did not use substances
H+V−S−: YWH with undetectable plasma HIV-1 who did not use substances
H+V−S+[M]: YWH with undetectable plasma HIV-1 who used marijuana alone
H+V−S+[M/T]: YWH with undetectable plasma HIV-1 used marijuana in combination with tobacco
YWOH: Youth without HIV-1
H–S−: Non-substance using YWOH
H–S+[M/T/A]: YWOH using a combination of marijuana, tobacco and alcohol
DEG: Differentially expressed genes
THC: Δ^9^-Tetrahydrocannabinol
THCA: Carboxy THC
CBD: Cannabidiol
HCA: Hierarchical clustering analysis
PCA: Principal component analysis
IPA: Ingenuity Pathway Analysis
VL: Viral load
IRB: Institutional review board
ACASI: Audio Computer-assisted Self-administered Interviewing
ELISA: Enzyme-linked immunosorbent assays
FDR: False discovery rate
FC: Fold change
CV: Coefficient of variation
