# Supplementary material for: Anti-inflammatory effects of recreational marijuana in virally suppressed youth with HIV-1 are reversed by use of tobacco products in combination with marijuana
Source: Retrovirology. 2022 May 31;19:10. doi: 10.1186/s12977-022-00594-4 (PMC9151353; doi:10.1186/s12977-022-00594-4)
Supplement: Supplementary file 1 — Additional file 1. Additional Figures S1 and S2. [file 12977_2022_594_MOESM1_ESM.pdf]

Additional file 1

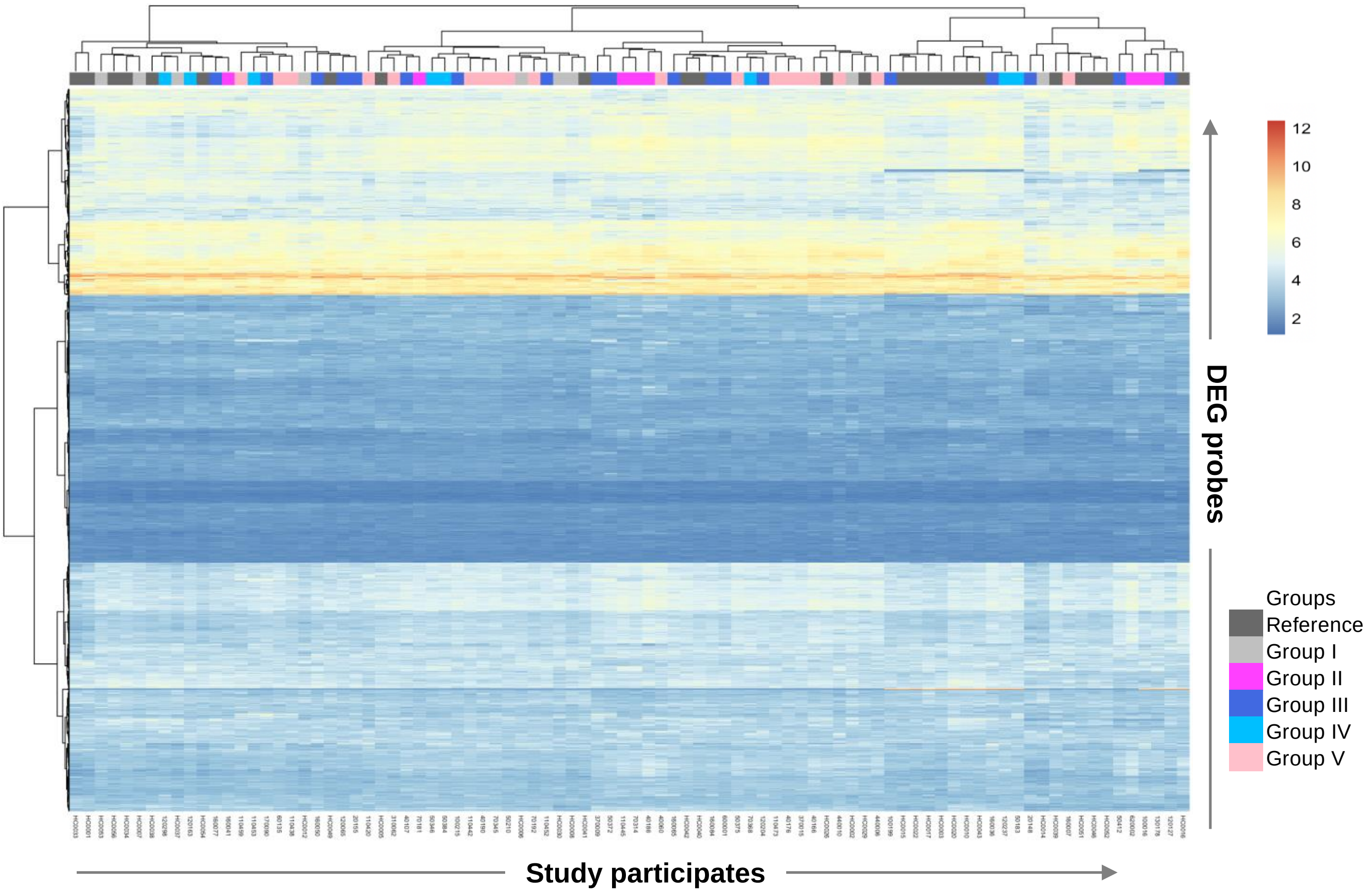

Figure S1. Hierarchical clustering of expression values among 88 study participants. Hierarchical clustering analysis was performed using the top 40% highly variable DEG probes (2,634 of 6,586) (right y axis, list of DEG available in supplemental material 2) representing the top 40% highly variable unique DEG (929 of 2,322) to predict the relatedness of the study groups (colored bars on top), including Reference Group (healthy nonsubstance use, n = 25, dark grey bars), Group I (healthy using a combination of alcohol, marijuana and tobacco, n = 9, light grey bars), Group II (virally unsuppressed nonsubstance-using YWH, n = 8, pink bars), Group III (virally suppressed nonsubstance-using YWH, n = 19, dark blue bars), Group IV (virally suppressed YWH using marijuana, n = 8, light blue bars), and Group V (virally suppressed YWH using marijuana plus tobacco, n = 19, light pink bars). Heatmap scales between 2 to 12 $\log_2$ gene expression values.

A.

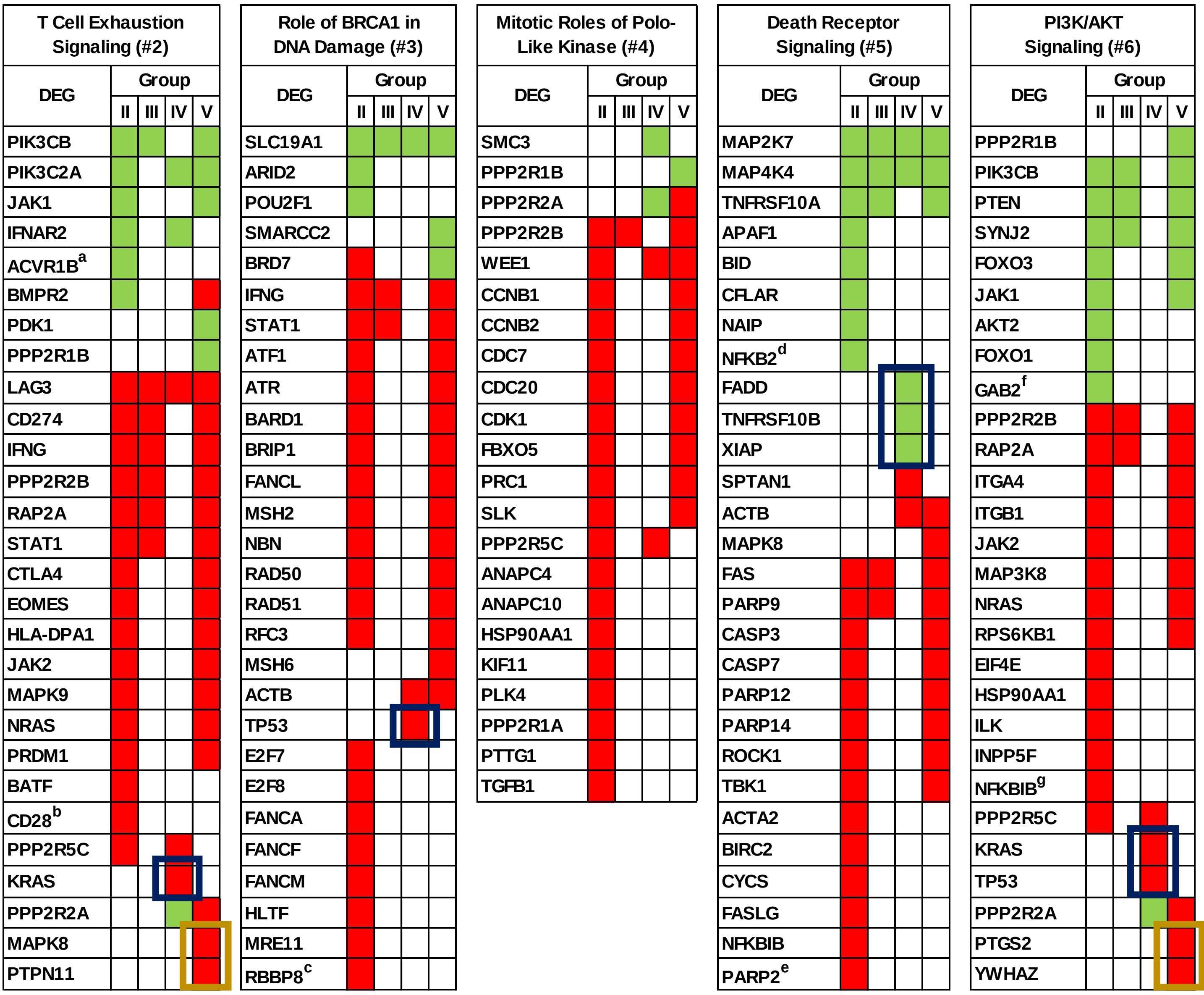

a. Additional Pathway #2 genes uniquely down-regulated in Group II [not shown]: AKT2, BCL6, FOXO1, FOXP1, IL6R, MAPK1, MGAT5, NFAT5 and PIK3CG.

b. Additional Pathway #2 genes uniquely upregulated in Group II [not shown]: HLA-DQA1, IL10RA, IL12RB1, IRF9, NFATC2, PDCD1, PIK3R3, PPP2R1A, PRKCQ, RAP1B, RRAS, RRAS2, SMAD3, STAT2, STAT4, TBX21, TGFB1 and TGFBR3.

c. Additional Pathway #3 genes uniquely upregulated in Group II [not shown]: RFC5 and SMARCE1.

d. Additional Pathway #5 genes uniquely down-regulated in Group II [not shown]: TNFRSF21 and TNFRSF25.

e. Addition Pathway #5 genes uniquely upregulated in Group II [not shown]: PARP3, PARP11 and PARP15.

f. Additional Pathway #6 genes uniquely down-regulated in Group II [not shown]: LIMS1, MAPK1, MDM2, NFKB2, PDPK1, PIK3CG, RAF1 and THEM4.

g. Additional Pathway #6 genes uniquely upregulated in Group II [not shown]: PIK3R3, PPP2R1A, RAP1B, RRAS, RRAS2, SOS1 and YWHAQ.

B.

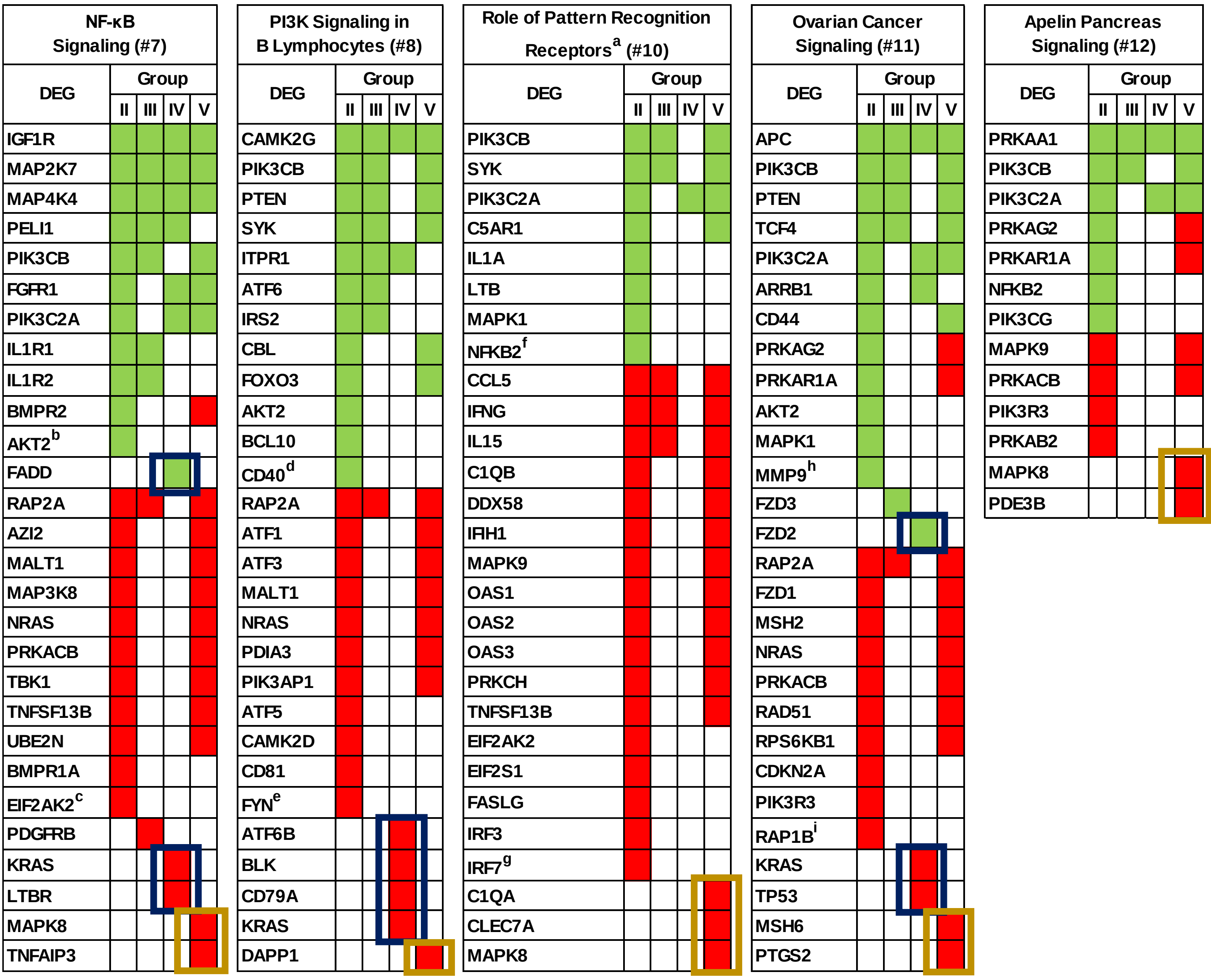

a: Role of Pattern Recognition Receptors in Recognition of Bacteria and Viruses

b: Additional Pathway #7 genes uniquely down-regulated in Group II [not shown]: BCL10, CD40, CREBBP, CSNK2A1, EP300, IGF2R, IL1A, MAP3K3, NFKB2, PIK3CG, PRKCZ, RAF1 and TLR6.

c: Additional Pathway #7 genes uniquely upregulated in Group II [not shown]: FGFR2, LCK, NFKBIB, PIK3R3, PRKCQ, RAP1B, RRAS, RRAS2, TAB3, TGFBR3 and TLR3.

d. Additional Pathway #8 genes uniquely down-regulated in Group II [not shown]: IL4R, MAPK1, NFAT5, NFKB2, PDPK1, PIK3CG, PPP3CA, PRKCZ, PTPRC, RAC1 and RAF1.

e. Additional Pathway #8 genes uniquely upregulated in Group II [not shown]: NFATC2, NFKBIB, PLEKHA3, PPP3CB, RAP1B, RRAS and RRAS2.

f. Additional Pathway #10 genes uniquely down-regulated in Group II [not shown]: PIK3CG, PRKCZ, TLR6 and TNFSF14.

g: Additional Pathway #10 genes uniquely upregulated in Group II [not shown]: PIK3R3, PRKCQ, TGFB1 and TLR3.

h: Additional Pathway #11 genes uniquely down-regulated in Group II [not shown]: PGF, PIK3CG and RAF1.

i: Additional genes Pathway #11 genes uniquely upregulated in Group II [not shown]: RRAS and RRAS2.

Figure S2. DEG in perturbed pathways. A. DEG in 5 pathways perturbed in both study Groups II and V. **B.** DEG in pathways perturbed uniquely in Group III [2 pathways, NF-κB Signaling (Pathway #7) and PI3K Signaling in B Lymphocytes (Pathway #8)] and Group V (Pathways #10, #11 and #12). Red-filled space: up-regulated gene; Green-filled space: down-regulated gene. Blue box: DEG specific to Group IV; Brown box: DEG specific to Group V.
